# Case Report: management of refractory glaucoma secondary to Sturge-Weber syndrome associated with ocular melanocytosis

**DOI:** 10.3389/fmed.2025.1604561

**Published:** 2026-01-05

**Authors:** Yan Zhou, Gangwei Cheng, Erqian Wang, Yang Zhang, Rongping Dai, Ailing Bian, Zaihong Weng

**Affiliations:** Department of Ophthalmology, Peking Union Medical College Hospital, Chinese Academy of Medical Sciences and Peking Union Medical College, Beijing, China

**Keywords:** refractory glaucoma, Sturge-Weber syndrome, ocular melanocytosis, class, trabeculotomy

## Abstract

**Background:**

Glaucoma associated with Sturge-Weber syndrome (SWS) is widely regarded as one of the most challenging types of secondary glaucoma, with the lowest surgical success rates. In this report, we present a successful management of refractory glaucoma secondary to Sturge-Weber syndrome accompanied by ocular melanocytosis.

**Case report:**

A 28-years-old female with a history of right eye glaucoma since childhood presented with poorly controlled intraocular pressure (IOP) despite multiple surgical interventions, including two trabeculectomies and one glaucoma drainage device implantation. She exhibited ipsilateral facial cutaneous port-wine stains and bluish-purple scleral pigmentation consistent with ocular melanocytosis (OM). Examination revealed a low corneal endothelial cell count and significant optic nerve atrophy (cup-to-disk ratio of 0.9) in the right eye. The patient underwent combined trabeculotomy- CO_2_ laser-assisted sclerectomy surgery (CLASS), which successfully reduced the IOP to below 10 mmHg with no complications and preserved visual acuity (VA).

**Conclusion:**

This case demonstrates the efficacy of combined trabeculotomy-CLASS in refractory glaucoma associated with SWS and ocular melanocytosis, offering a promising surgical alternative for complex cases.

## 1 Introduction

Sturge-Weber Syndrome (SWS) is a congenital neurocutaneous disorder characterized by facial capillary malformations (port-wine stains), leptomeningeal angiomatosis, and glaucoma (1). Among the ocular manifestations of SWS, ocular melanocytosis (OM) or its variant involving the skin, oculodermal melanocytosis (ODM, also known as nevus of Ota), is exceedingly rare (2–5). The literature on optimal management strategies for such cases remains limited, rendering both diagnosis and treatment particularly challenging. This case report describes a patient with refractory glaucoma associated with SWS and OM, and introduces a novel surgical approach–combined trabeculotomy and CO_2_ laser-assisted sclerectomy surgery (CLASS) as an effective solution for managing refractory glaucoma in such complex cases.

## 2 Case report

A 28-years-old female patient, born in 1997, was initially noted to have elevated intraocular pressure (IOP) in the right eye at an external hospital in 2001, leading to a diagnosis of unilateral glaucoma. Surgical interventions included trabeculectomy performed in 2003 and again in 2006, followed by implantation of a glaucoma drainage device in 2007. Since 2013, her right eye IOP has been managed with topical antiglaucoma medications (brinzolamide and travoprost), maintaining IOP at approximately 20 mmHg. In March 2024, she presented to the Ophthalmology Department of Peking Union Medical College Hospital, where additional IOP-lowering agents (carteolol and brimonidine) were introduced, achieving IOP control within the range of 25–30 mmHg.

**FIGURE 1:**
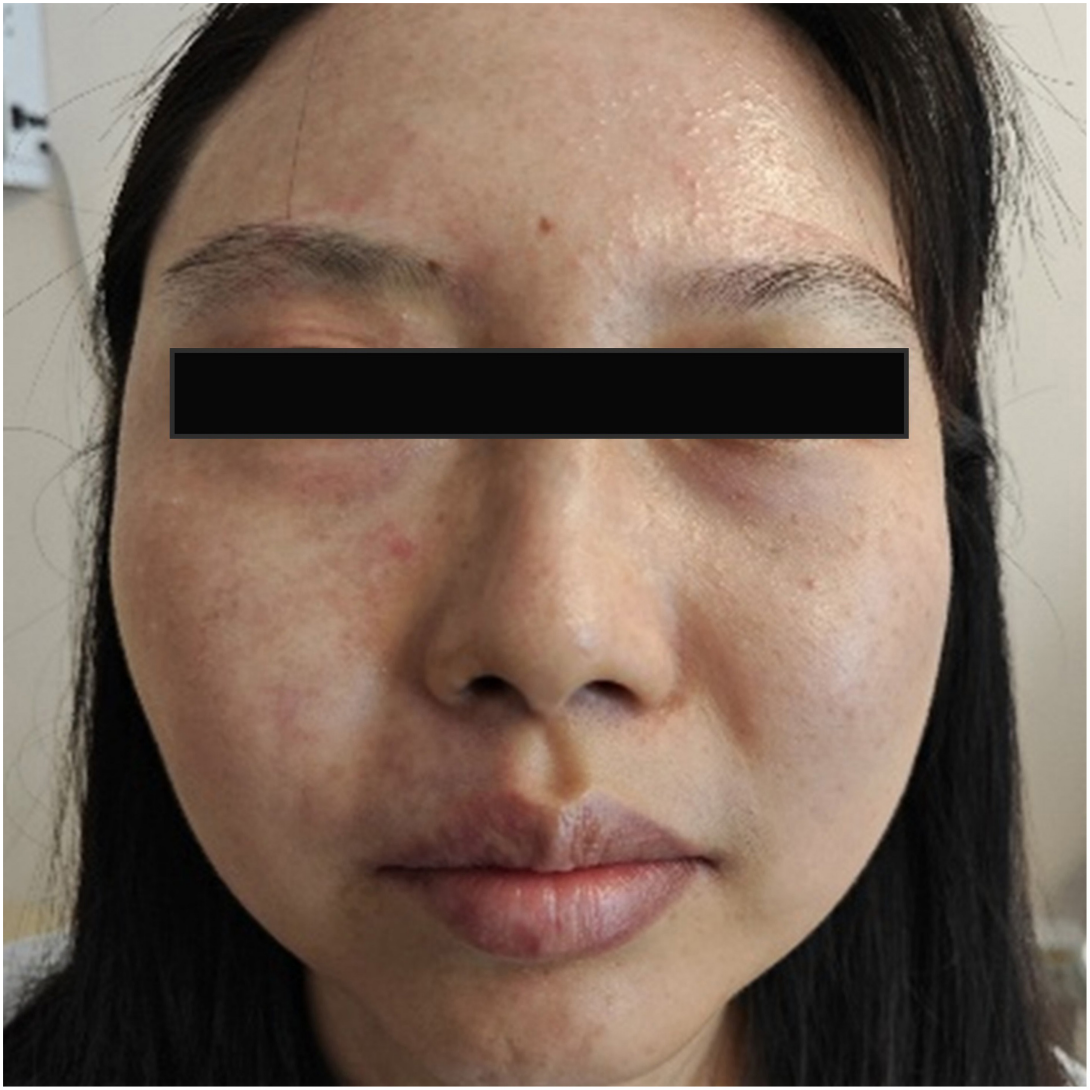
Facial photograph showing diffuse, faint erythematous patches on the right side of the face, consistent with a port-wine stain characteristic of Sturge-Weber syndrome.

The patient has bilateral myopia, with a refractive error of approximately −6.5 diopters (DS) in the right eye and −4.5 DS in the left eye. Since birth, she has exhibited a cutaneous erythematous patch on the right side of her face and neck, which has undergone multiple cosmetic laser treatments (Figure 1). The neck patch had almost disappeared. No history of developmental delay, headaches, seizures, or neurological symptoms was solicited. She denied any other systemic diseases or family history of similar conditions.

**FIGURE 2:**
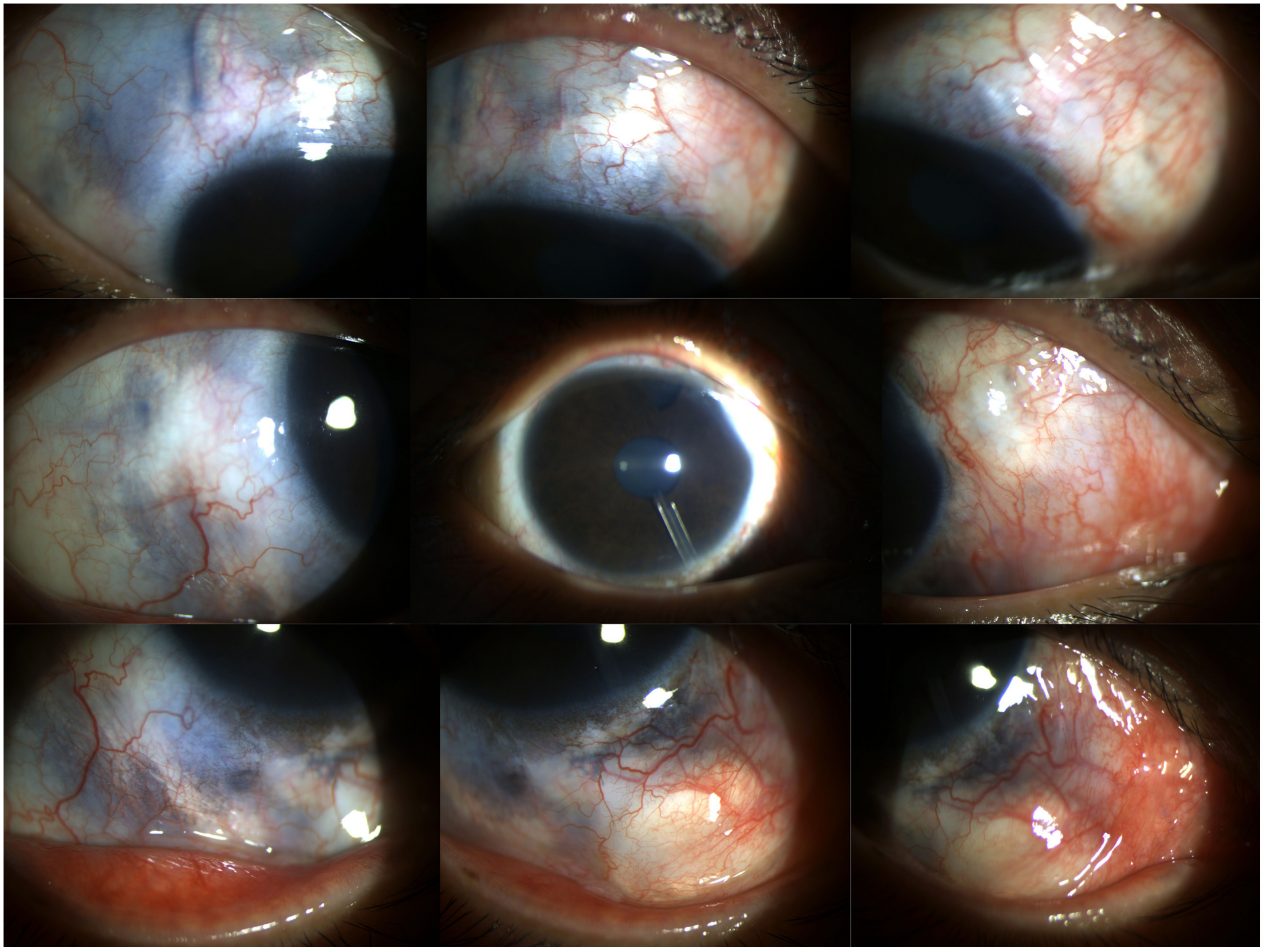
Anterior segment photograph of the right eye reveals engorged conjunctival vessels, bluish-purple scleral pigmentation indicative of ocular melanocytosis, a flat filtering bleb located superiorly and superonasally, and a deep anterior chamber with visible inferonasal drainage tube and patent superonasal peripheral iridotomy.

**FIGURE 3:**
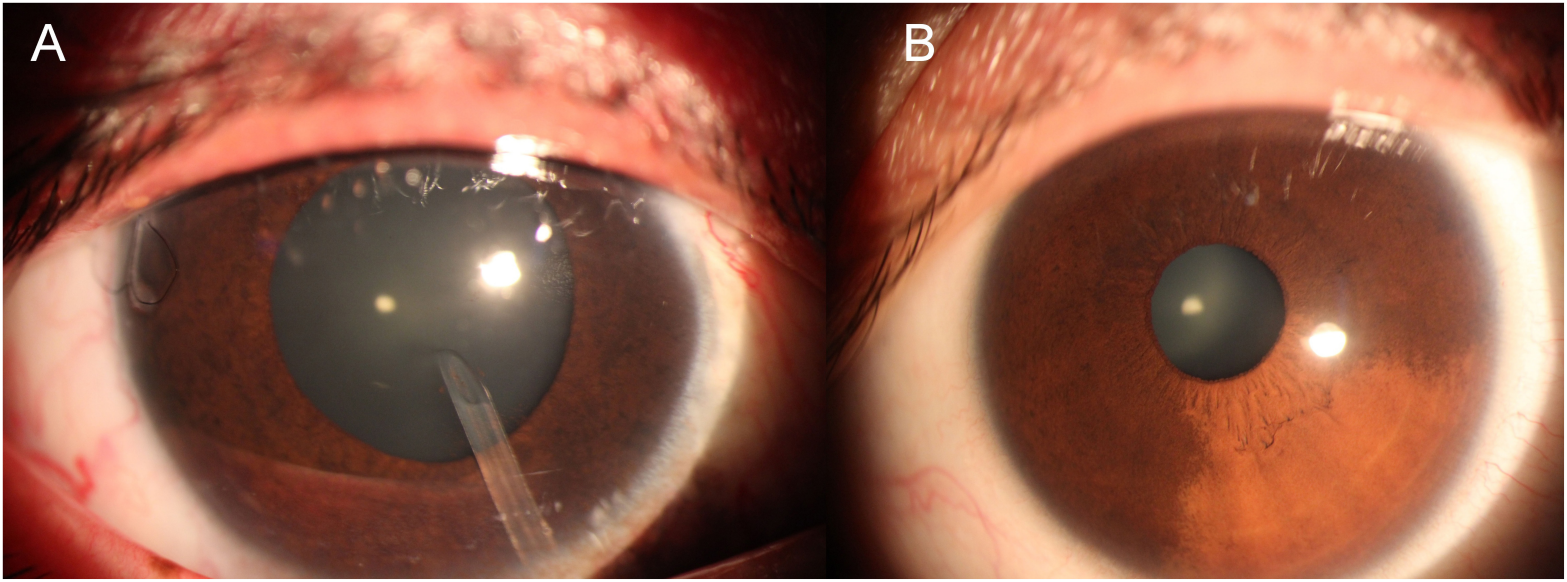
Comparative anterior segment images demonstrating iris pigmentation. **(A)** Right eye with scattered iris pigmentation; **(B)** left eye showing extensive iris pigmentation. Both eyes exhibit sparse iris texture, with more pronounced pigmentation and heterochromia in the right eye affected by ocular melanocytosis.

On examination, a diffuse, faint red patch was observed on the right side of her face (Figure 1). Corrected visual acuity (VA) was 0.6 in both eyes. Non-contact tonometry revealed an IOP of 25 mmHg in the right eye and 20 mmHg in the left eye. The right eye exhibited conjunctival and scleral pigmentation, with a distinct bluish-purple discoloration of the sclera, particularly prominent in the superotemporal region (Figure 2). The right iris demonstrated scattered pigmentation, while the left iris showed extensive pigmentation, and the iris texture of both eyes was obviously sparse (Figure 3). These findings are highly keeping with OM. In the right eye, a glaucoma drainage tube was observed inferonasally, with a localized scleral elevation suggesting prior allograft scleral transplantation. A broad peripheral iridotomy was noted superonasally, and gonioscopy revealed two areas of peripheral anterior synechiae (PAS) in the same quadrant, indicative of prior trabeculectomy sites. The angle exhibited a grade II pigmentation according to the Scheie classification. The corresponding conjunctival filtering bleb appeared flat. The right optic disk was pale, with a cup-to-disk ratio (CDR) of 0.9, and no choroidal hemangioma-like changes were detected. The left optic disk was normal. B-scan ultrasonography and optical coherence tomography (OCT) showed no evidence of choroidal thickening or other structural abnormalities in either eye. Endothelial cell counts were 1425.8 cells/mm^2^ in the right eye and 3138.3 cells/mm^2^ in the left eye.

## 3 Discussion

The patient presents with an initial impression of SWS combined with OM. SWS is a rare, congenital neurocutaneous disorder characterized by facial capillary malformations (port-wine stains or naevus flammeus), leptomeningeal angiomatosis, and glaucoma (1). The most common ocular manifestations of SWS include glaucoma and choroidal hemangioma. Additionally, patients may exhibit vascular lesions on the eyelid, abnormal vascular lesions of the conjunctiva, episcleral hemangiomas, iris heterochromia, retinal occlusion, and iris mammillations (6). In rare cases, SWS may coexist with OM or ODM (2–5). Notably, the clinical features in this patient may overlap with another rare neurocutaneous syndrome, phakomatosis pigmentovascularis (PPV), which is characterized by the coexistence of congenital pigmented nevi and cutaneous vascular malformations (1, 7, 8). In PPV, the cutaneous vascular malformations typically present as naevus flammeus, while the pigmented lesions may include OM or ODM (9, 10). Although glaucoma has been less frequently reported in PPV patients, this finding suggests that these two conditions may share common pathogenic mechanisms (8, 11).

To preserve the visual function of the patient’s right eye, it is imperative to perform another anti-glaucoma surgery. The right eye, having undergone multiple anti-glaucoma procedures and now in the advanced stage of glaucoma, presents significant challenges in terms of surgical site selection and technique. Cyclophotocoagulation was first excluded because the patient is a young female, and photocoagulation could lead to irreversible eyeball atrophy, eliminating future treatment opportunities. Additionally, revision of the existing filtering bleb was deemed unsuitable due to significant scleral scarring and conjunctival thinning at previous surgical sites, combined with observed peripheral anterior synechiae in the superior and superonasal quadrants on gonioscopy, which posed substantial risks of surgical failure and potential perforation/fistula formation. The prevailing view suggests that early-onset glaucoma in SWS is primarily associated with anterior chamber angle abnormalities, while late-onset glaucoma is predominantly linked to elevated episcleral venous pressure (EVP) (12, 13). Concurrent OM may contribute to increased IOP due to pigment deposition in the trabecular meshwork and elevated aqueous outflow resistance caused by melanocytes (3, 9). Repeating angle-based surgeries such as goniotomy or trabeculotomy may fail to address the underlying issue of elevated distal EVP, leading to surgical failure. We also ruled out other minimally invasive glaucoma surgeries (MIGS), not only because of the need for sufficient IOP reduction but also because, after multiple previous filtering surgeries, the patient’s natural drainage pathways may have atrophied, making external drainage procedures that rely on the ocular surface high-risk for failure. Moreover, the patient’s low corneal endothelial cell count makes another drainage device implantation untenable. Filtration surgery carries significant risks, including expulsive choroidal hemorrhage, bleeding, and high rates of bleb failure. Although non-penetrating deep sclerectomy (NPDS) has demonstrated efficacy comparable to trabeculectomy in managing SWS-related glaucoma, the presence of episcleral hemangioma and angle anomalies increases the surgical complexity and failure rate (13). A retrospective study published in 2023 demonstrated that combined trabeculotomy and non-penetrating deep sclerectomy (CTNS) significantly reduces IOP in patients with secondary glaucoma associated with SWS presenting severe episcleral vascular malformations, compared to traditional filtering procedures or glaucoma drainage device implantation (14). In this case, the patient had previously undergone trabeculectomy at the superior and nasal-superior quadrants and drainage device implantation at the nasal-inferior quadrant, which are not conventional or easily accessible surgical sites. We have reasonable concerns regarding the ocular surface health in the remaining surgical site–the temporal-superior quadrant. Significant episcleral pigmentation in this area poses a high risk of unforeseen complications. Given that no abnormal vessels were observed in the right eye during gonioscopy, and based on our team’s extensive experience with combined trabeculotomy-CO_2_ laser-assisted sclerectomy surgery (CLASS) and our expertise in managing refractory glaucoma, we ultimately opted for a combined trabeculotomy-CLASS procedure. The use of CO_2_ laser energy enables precise ablation of dry tissue, which is absorbed by fluid. When aqueous humor percolates through, the laser’s effect ceases, thereby avoiding perforation and reducing the technical difficulty associated with manual dissection (15). This approach focuses on deep drainage while incorporating superficial outflow pathways, effectively reducing intraocular pressure while minimizing the risks associated with superficial tissue damage.

**FIGURE 4:**
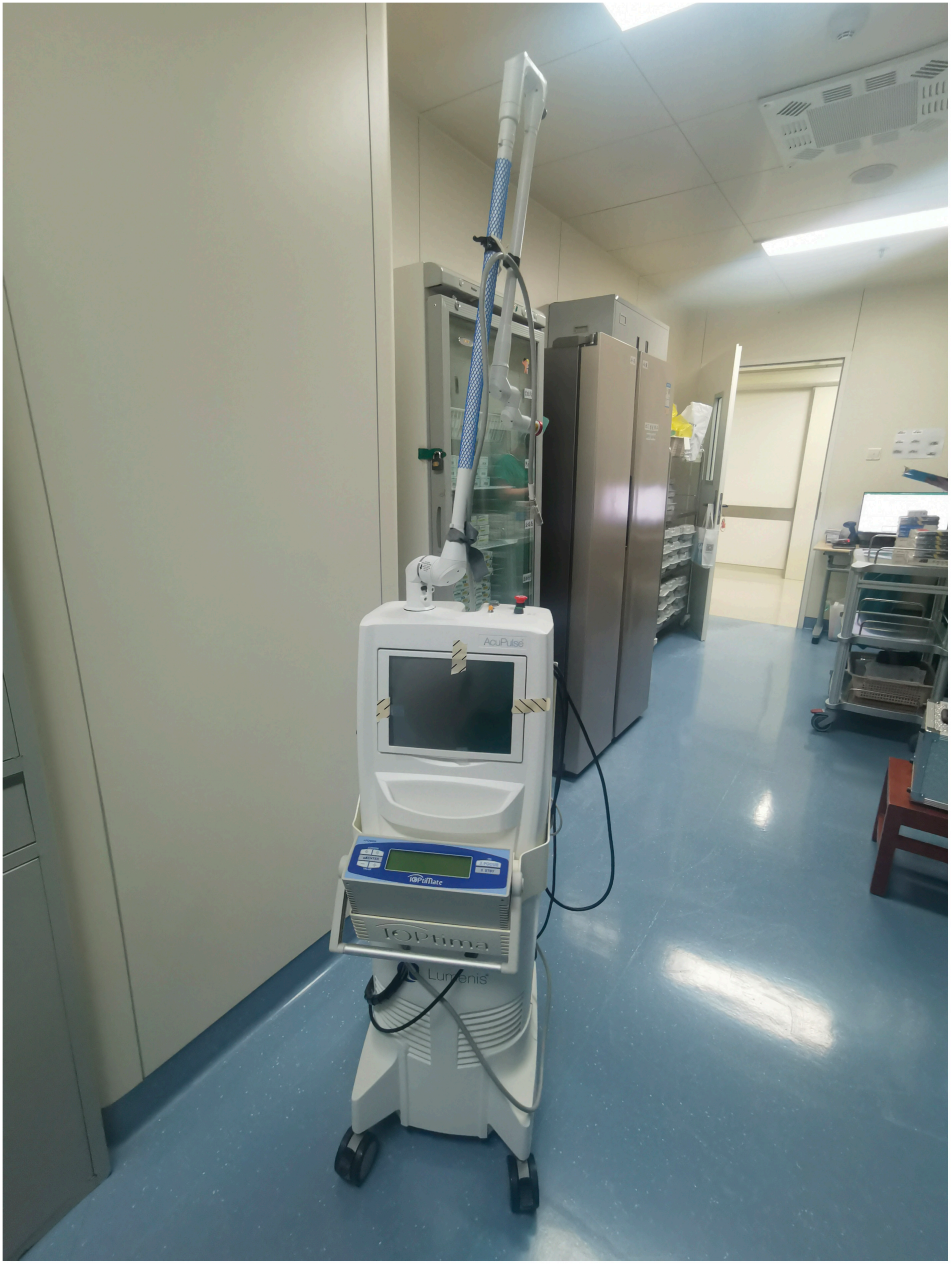
The Lumenis AcuPulse^®^ CO_2_ laser system with IOPtiMate™ surgical adapter (IOPtima Ltd.) used in the combined trabeculotomy-CLASS procedure.

During the surgery, a 5 mm × 5 mm rectangular scleral flap, approximately half-thickness, was created. Mitomycin C (0.4 mg/mL) soaked sponges were applied beneath and above the scleral flap for 240 s and under the conjunctiva for 30 s, followed by extensive irrigation with balanced salt solution. A CO_2_ laser (Figure 4) set at 21 W was used to create a 4 mm × 2.4 mm scleral pool with an ablation depth of approximately 80%. Mitomycin C (0.4 mg/mL) was reapplied to the scleral pool for 150 s, followed by thorough irrigation. A trabeculectomy measuring approximately 1 mm × 1.5 mm was performed. The scleral flap was secured using both fixed and adjustable sutures. The postoperative regimen included topical atropine for cycloplegia, prednisolone acetate (1%) for anti-inflammatory therapy, and ofloxacin eye drops for antibiotic prophylaxis. Close monitoring of the ocular condition was maintained, with targeted postoperative care and selective removal of adjustable sutures as needed.

The presence of scleral pigmentation and abnormal vascularity presents specific challenges for surgeries. Potential risks include: (1) technical difficulty in creating a uniform and adequately deep scleral flap due to altered tissue architecture; (2) increased risk of intraoperative bleeding from abnormal episcleral vessels, potentially compromising surgical visualization; and (3) possible enhanced postoperative fibrosis of the external filtration site due to melanocyte-rich tissue, potentially reducing long-term efficacy. Whenever feasible, selecting a surgical site that minimizes exposure to heavily pigmented or vascularly anomalous areas is advisable. However, in this case, the previous surgeries limited our options for alternative surgical placement. Both during surgery and in the postoperative course, we were fortunate not to encounter these potential complications.

During the procedure, prominent conjunctival and superficial scleral vasculature was observed, yet hemostasis was successfully achieved. The scleral flap was created, avoiding areas with the heaviest pigmentation. Minimal scleral pigmentation was noted within the deeper layers. The scleral tissue was stiffer and less elastic compared to healthy individuals. A small amount of light bloody fluid was seen exuding from the interlamellar sclera, suggesting abnormal scleral vascular development. Despite these challenges, the surgical procedure was completed successfully, and no complications such as choroidal detachment or suprachoroidal hemorrhage were observed during the perioperative period. Postoperative management included targeted early-phase care. From postoperative day 1 through week 3, during which all adjustable sutures were removed, the IOP in the right eye remained between 5 and 9 mmHg. Topical anti-inflammatory medication was gradually tapered to a low-dose steroid maintenance regimen. Specific IOP measurements were documented at key intervals: 8 mmHg at 2 months, 9 mmHg at 3 months, and stable between 10 and 11 mmHg from the fourth month to the most recent follow-up at 15 months. Beginning at 10 months postoperatively, the patient performed digital ocular massage twice daily (morning and evening) to maintain filtration. No other interventions such as bleb needling were required. Corrected VA has been maintained between 0.6 and 0.8, with the latest measurement being 0.8 at the 15-months visit. IOP remained stable at 11 mmHg at the 15-months follow-up. Visual field testing performed at 1 year postoperatively showed no significant progression compared to preoperative results, confirming functional stability.

Glaucoma caused by SWS is widely regarded as one of the most challenging forms of secondary glaucoma to manage surgically, with historically low success rates. While goniotomy or trabeculotomy are often considered as initial surgical interventions to address trabecular meshwork resistance, they do not adequately address elevated EVP–a key pathophysiological factor in SWS. Although relatively low-risk, these procedures are associated with high failure rates (16). Various other approaches, including drainage device implantation, trabeculectomy, and combined trabeculotomy-trabeculectomy, have been employed with varying success; however, accompanying complications such as hypotony, choroidal effusion, or hemorrhage are not uncommon (2, 12, 13). Non-penetrating deep sclerectomy alone has also been considered insufficient to address severe episcleral hemangiomatosis and angle dysgenesis. Notably, a 2023 study first reported the combination of trabeculotomy with non-penetrating deep sclerectomy (CTNS) in an SWS glaucoma patient with severe episcleral vascular malformation, achieving satisfactory IOP reduction and safety (14). Cyclodestructive procedures have been used in buphthalmic cases. Of particular relevance to this case, SWS associated with OM, only isolated cases treated with trabeculectomy have been reported, with limited follow-up (5, 8, 11). To our knowledge, this is the first reported case employing a combined trabeculotomy and CO_2_ laser-assisted sclerectomy surgery (CLASS) technique in a patient with both SWS and OM. This approach not only addresses trabecular and EVP-related mechanisms simultaneously but also demonstrates that deep, non-penetrating surgery may be feasible and effective in such complex phenotypes, challenging conventional surgical assumptions.

This outcome leads us to believe that combined trabeculotomy and CLASS may also be highly effective in cases of glaucoma secondary to SWS alone or OM alone. However, the generalizability of this technique requires careful anatomical and surgical considerations. Key factors include the extent of episcleral vascular abnormalities, the degree of angle involvement, and previous surgical history. In cases with extensive episcleral hemangiomas or severe scleral thinning, modified surgical techniques may be necessary. Additionally, the absence of abnormal vessels in the surgical quadrant appears to be an important anatomical prerequisite for considering this approach. While our results are promising, further validation through larger studies and longer follow-up periods is needed to establish the broader applicability of this technique across the spectrum of SWS and OM related glaucomas. Anyway, minimally invasive deep drainage procedures may represent a promising direction for future advancements in the management of this complex condition.

## Data Availability

The original contributions presented in this study are included in this article/supplementary material, further inquiries can be directed to the corresponding author.
